# Oral Cell Lysates Reduce the Inflammatory Response of Activated Macrophages

**DOI:** 10.3390/jcm12041701

**Published:** 2023-02-20

**Authors:** Layla Panahipour, Azarakhsh Oladzad Abbasabadi, Reinhard Gruber

**Affiliations:** 1 Department of Oral Biology, University Clinic of Dentistry, Medical University of Vienna, Sensengasse 2a, 1090 Vienna, Austria; 2 Department of Periodontology, School of Dental Medicine, University of Bern, 3010 Bern, Switzerland; 3 Austrian Cluster for Tissue Regeneration, 1200 Vienna, Austria

**Keywords:** inflammation, necrosis, cell lysates, oral cells, macrophages, bioassay, gingival fibroblasts, oral epithelial cells

## Abstract

Necrotic cell damage occurs as a consequence of invasive dental procedures. Loss of membrane integrity being the hallmark of necrotic cells leads to the release of cytoplasmic and membranous components. Macrophages are predestined to respond to lysates originating from necrotic cells. Here, we implement necrotic lysates from human gingival fibroblasts, HSC2, and TR146 oral epithelial cell lines, and RAW264.7 macrophage cell lines to be tested for their potential to modulate the inflammatory response of macrophages. To this aim, necrotic cell lysates were prepared by sonication or freezing/thawing of the respective cell suspension. Necrotic cell lysates were tested for their potential to modulate the lipopolysaccharide (LPS)-induced expression of inflammatory cytokines using RAW264.7 macrophages as a bioassay. We show here that all necrotic cell lysates, independent of the origin and the preparation way, reduced the expression of IL1 and IL6 in LPS-induced RAW264.7 macrophages, most obviously shown for TR146 cells. This finding was supported in a bioassay when macrophages were exposed to poly (I:C) HMW, an agonist of TLR-3. Consistently, all necrotic lysates from gingival fibroblasts, HSC2, TR146, and RAW264.7 cells reduced the nuclear translocation of p65 in LPS-exposed macrophages. This screening approach supports the overall concept that necrotic cell lysates can modulate the inflammatory capacity of macrophages.

## 1. Introduction

Accidental cell death or primary necrosis is not a physiological process, but may occur as a consequence of severe insults of physical (e.g., high pressures, temperatures, or osmotic forces), chemical (e.g., extreme pH variations), or mechanical (e.g., shear forces) stress, which prompts an immediate rupture of the cell membrane and release of intracellular molecules [1,2]. In dentistry, cell damage occurs upon drilling and the insertion of dental implants, where it is particularly the dying osteocytes being affected by the invasive procedures [3,4]. Surgical techniques associated with implant dentistry, such as harvesting of autologous bone [5] or soft tissue complications and grafting [6,7], may also cause cell damage. Even though not explicitly shown, nonsurgical periodontal procedures, such as scaling and root planing, can damage oral epithelial cells and fibroblasts of the underlying connective tissue [8]. Electrosurgery [9] and cryosurgery [10] are further examples of how a dental procedure may cause necrotic cell damage. Even though there is causality that invasive dental procedures may damage local cells, histological evidence of necrotic cell damage is rare. 

Cell damage by primary necrosis is a side effect of tremendous mechanical and thermal stress rather than a controlled biological process such as apoptosis [11], necroptosis [12], and pyroptosis [13]. Moreover, apoptosis, necroptosis, or pyroptosis are all under the control of caspases that are either activated or suppressed, thereby initiating a cascade of events that dictate cell fate—the mode of which cells die. Thus, there are molecular markers that can distinguish and, therefore, identify apoptosis [11], necroptosis [12], and pyroptosis [13]. With apoptosis, the activation of caspase-3 and caspase-7 is considerably enhanced in the gingival tissue of patients with periodontitis [14]. For necroptosis, elevated levels of receptor-interacting protein serine-threonine kinases-1 (RIPK1), phosphorylated RIPK3, mixed lineage kinase domain-like protein (MLKL), and phosphorylated MLKL were observed in gingival tissues collected from patients with untreated chronic periodontitis [15]. Concerning pyroptosis, NLRP3, caspase-1, caspase-4, and IL18 were more pronounced in the inflammatory gingiva compared to healthy gingiva [16,17]. However, such signaling cascades are not activated when mechanical and thermal damage impair the integrity of the cell membrane. Necrotic cells immediately release the content of their cytoplasm and membrane fragments into the local environment, signaling the need for repair. It is, therefore, hard to identify necrotic cells in a histological section; hence, it is difficult to study the distribution and function of necrotic cells in vivo. Moreover, considering that necrosis is not a physiological cellular event, the cellular response of vital cells to necrotic cells is unpredictable. 

One strategy to access this research question is to take advantage of in vitro settings where necrotic cell lysates are produced in a controlled and reproducible way. For instance, necrotic cells prepared by freezing/thawing release chromatin associated IL1 that causes acute inflammation, particularly by the recruitment of neutrophils and macrophages [18]. Necrotic cell lysates prepared by repeated freezing/thawing of HEK-293 cells release clusterin [19], a chaperone known for triggering the release of inflammatory mediators by macrophages [20,21]. Moreover, uric acid is considered an endogenous danger signal [22] and sodium urate crystals can provoke an inflammatory response of RAW264.7 macrophages [23]. In contrast, however, extracellular vesicles released from damaged mesenchymal cells can prevent the M1 polarization in LPS-stimulated RAW264.7 macrophages [21,22]. This aspect supports our recent observations that lysates prepared from necrotic murine bone marrow stromal ST2 and the osteocytic cell line IDG-SW3 are polarization inhibitors of LPS-induced RAW264.7 macrophages [24]. Thus, necrotic cell lysates are potential modulators of inflammatory activity. 

The present in vitro study extends previous work on ST2 and IDG-SW3 cells [2] by implementing necrotic lysates from human gingival fibroblasts, HSC2 and TR146 oral epithelial cell lines, and RAW264.7 macrophage cell lines to be tested for their potential to modulate a simulated inflammatory response of macrophages. Cell lysates were prepared using sonication and freezing/thawing cycles. The inflammatory response of macrophages was tested with LPS and poly (1:C) HMW-exposed RAW264.7 macrophages. Based on this in vitro setting, we intended to study how damaged oral cells modulate the polarization of macrophages. 

## 2. Methods

### 2.1. Cell Lines

Human gingival fibroblasts were prepared from small pieces of gingiva that were obtained during wisdom tooth removal surgery from three healthy donors who gave their informed consent. The Ethical Committee of the Medical University of Vienna approved the protocol (EK Nr. 631/2007). Pieces of gingiva were kept in a tissue culture flask allowing fibroblast outgrowth, followed by their expansion through repeated passaging. Fibroblasts had the expected spindle-shape morphology. The oral squamous cell carcinoma cell line HSC2, originally obtained from the Health Science Research Resources Bank (Sennan, Japan), was kindly provided by Prof. Rausch-Fan, Department of Periodontology, Medical University of Vienna, Austria. The oral squamous cell carcinoma cell line TR146 from the European Collection of Authenticated Cell Cultures was shared with Winfried Neuhaus from the Competence Unit Molecular Diagnostics, Center for Health and Bioresources, Austrian Institute of Technology GmbH. RAW264.7 macrophage-like cells were from the American Type Culture Collection (LGC Standards GmbH, Wesel, Germany). All cells were cultured in a humidified atmosphere at 37 °C in a growth medium consisting of DMEM, 10% fetal calf serum (FCS), and 1% antibiotics (Invitrogen Corporation, Carlsbad, CA, USA). RAW264.7 macrophage-like cells were expanded in growth medium only and seeded at 1 × 10^6^ cells/cm^2^ into 24-well plates; cells were treated with lipopolysaccharide from *Escherichia coli* 0111: B41 (LPS; Sigma Aldrich, St. Louis, MO, USA) at 100 ng/mL and poly (1:C) HMW (InvivoGen, Toulouse, France) at 10 µg/mL in the presence and absence of the necrotic cell lysates overnight (16–18 h). 

### 2.2. Cell Lysates

Gingival fibroblasts, HSC2, TR146, and RAW264.7 cells were suspended at 4 × 10^6^ cells/mL in Dulbecco’s modified essential medium (DMEM) supplemented with 10% FCS and antibiotics (all Invitrogen, Grand Island, NY, USA) and subjected to: (i) sonication for three times each time 15 s (Sonoplus; Bandelin electronic GmbH & Co. KG; Berlin, Germany); or (ii) three times freezing/thawing for 8 min at −80 °C and room temperature. The necrotic cell lysates underwent centrifugation at 2600 RCF for 5 min (5420; Eppendorf SE, Hamburg, Germany). All supernatants, now considered as necrotic cell lysate, were prepared fresh for each independent experiment.

### 2.3. Reverse Transcription Quantitative Real-Time PCR (RT-qPCR)

Total RNA was extracted using the ExtractMe total RNA kit (Blirt S.A., Gdańsk, Poland). Next, complementary DNA (cDNA) was synthesized through reverse transcription of the total RNA (LabQ, Labconsulting, Vienna, Austria). The polymerase chain reaction was performed (LabQ, Labconsulting, Vienna, Austria) on a CFX Connect™ Real-Time PCR Detection System (Bio-Rad Laboratories, Hercules, CA, USA). Sequences of the primers were IL1-F: TTGGTTAAATGACCTGCAACA, IL1-R: GAGCGCTCACGAACAGTTG; IL6-F: GCTACCAAACTGGATATAATCAGGA, IL6-R: CCAGGTAGCTATGG-TACTCCAGAA; GAPDH-F: AACTTTGGCATTGTGGAAGG, GAPDH-R: GGATGCAGGGATGATGTTCT; the amount of each specific mRNA was normalized to the housekeeping gene GAPDH using the ^ΔΔ^Ct method. RT-qPCR data are depicted compared to the unstimulated control, which was assumed as 1.0 in all the analyses. 

### 2.4. Immunoassay

RAW264.7 macrophage-like cells were treated with LPS and poly (1:C) HMW in the presence and absence of the necrotic cell lysates for 16–18 h. Supernatants were collected and centrifugated prior to storing at −20 °C for not more than three weeks. The concentration of IL6 in the supernatant was measured by immunoassay according to the manufacturer’s instruction (DY406, R&D Systems, Minneapolis, MN, USA). The immunoassay is a solid phase sandwich ELISA and the binding of the detection antibody linked to Streptavidin-HRP was identified by the colorimetric substrate tetramethylbenzidine (T0440, Sigma). 

### 2.5. Immunofluorescent Analysis

The immunofluorescent examination of the nuclear translocation of NFκB-p65 was implemented in RAW264.7 cells seeded into Millicell^®^ EZ slides (Merck KGaA, Darmstadt, Germany) at 1 × 10^6^ cells/cm^2^. Cells were serum-deprived overnight before being treated with 100 ng/mL LPS in the presence and absence of the necrotic cell lysates for another 30 min. The cells were fixed using 4% paraformaldehyde and blocked with 1% bovine serum albumin (BSA, Sigma Aldrich, St. Louis, MO, USA). The anti-NF-κB p65 antibody (IgG, 1:800, Cell Signaling Technology, CST, Cambridge, UK, #8242) was added to the cells at 4 °C for 16 h, followed by the goat anti-rabbit Alexa 488 (1:1000, CST, #4412) and Fluoromount-GTM containing DAPI (Invitrogen, Carlsbad, CA, USA). Images were taken using revolve fluorescent microscope (Echo, Bico, San Diego, CA, USA). DAPI blue nuclear staining was quantified by ImageJ software (NIH, Bethesda, MA, USA).

### 2.6. Statistical Analysis

The experiments were repeated at least three times. Statistical analysis was based on an uncorrected Friedmann test using Prism v8 (GraphPad Software, La Jolla, CA, USA). The comparison was between the LPS group and its combination with each single cell lysate. *p*-values are reported.

## 3. Results

### 3.1. Necrotic Cell Lysates Reduce LPS-Induced IL1 and IL6 in RAW264.7 Cells

**Figure 1:**
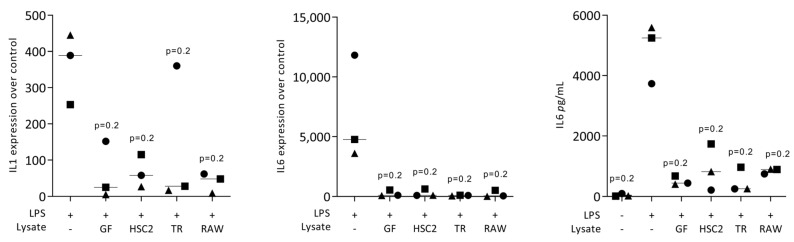
Sonicated necrotic cell lysates reduce LPS-induced cytokine expression of RAW264.7 macrophages. RAW264.7 macrophages exposed to LPS with and without sonicated cell lysates from gingival fibroblasts (GF), HSC2, TR146 (TR), and RAW264.7 (RAW) cells to induce the expression of IL1 and IL6 and the release of IL6 protein into the supernatant. The expression changes were normalized to an unstimulated control, while in the supernatant the basal IL6 production is shown. The dots represent three independent experiments. Statistical analysis was based on a Friedmann test and *p*-values are indicated.

**Figure 2:**
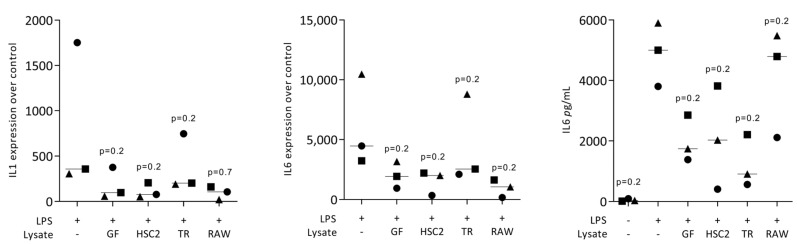
Freezing/thawing cell lysates reduce LPS-induced cytokine expression of RAW264.7 macrophages. RAW264.7 macrophages exposed to LPS with and without freezing/thawing cell lysates from gingival fibroblasts (GF), HSC2, TR146 (TR), and RAW264.7 (RAW) to induce the expression of IL1 and IL6 and the release of IL6 protein into the supernatant. The expression changes were normalized to unstimulated control. In the supernatant, the basal IL6 production is shown. The dots represent the three independent experiments. Statistical analysis was based on a Friedmann test and *p*-values are indicated.

**Figure 3:**
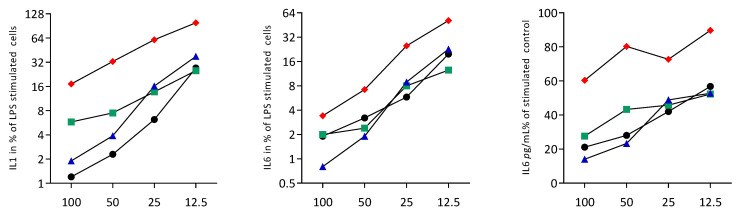
Dose-response of sonicated necrotic cell lysates reduce cytokine expression in RAW264.7 cells. RAW264.7 macrophages were exposed to LPS with and without different percentages of sonicated cell lysates (12.5, 25, 50, and 100%) from gingival fibroblasts (black), HSC2 (green), TR146 (blue), and RAW264.7 (red). The percentage of LPS-stimulated control expression (100%) is shown. The dot plots represent the mean of two independent experiments.

To rule out any cytotoxic effects of cell lysates, we performed an MTT assay. When RAW264.7 macrophages were exposed to the necrotic lysates from fibroblasts, HSC2, TR146, and RAW264.7 cells, no viability changes were noticed We then assessed the impact of the necrotic cell lysates on LPS-induced inflammation of RAW264.7 cells. RT-PCR analyses of IL1 and IL6, and an immunoassay of IL6 were conducted. LPS caused the expected increased expression of IL1 and IL6 at the transcriptional level, and of IL6 at the protein level. This LPS response was lowered by the presence of the necrotic cell lysates prepared by sonication (Figure 1) and freezing/thawing (Figure 2). Dose-response experiments revealed that sonicated lysates prepared from gingival fibroblasts, HSC2 and TR146, can be diluted up to 10-fold, maintaining their anti-inflammatory activity (Figure 3). However, once cell lysates from RAW264.7 cells are diluted, they lose their anti-inflammatory activity (Figure 3). To rule out that necrotic cell lysates hinder RAW264.7 cells from being exposed to LPS, we pre-exposed cells with LPS prior to adding TR146 cell lysates prepared by sonication. Furthermore, in this setting, TR146 cell lysates reduced the LPS-induced expression of IL1 and IL6 in RAW264.7 cells (Supplementary Figure S1). The lysates alone could not increase cytokine expression, there was even a modern decrease in basal IL6 expression (Supplementary Table S1).

### 3.2. Sonicated Cell Lysates Reduce TLR3 Agonist-Induced Cytokine Expression in RAW264.7 Cells

**Figure 4:**
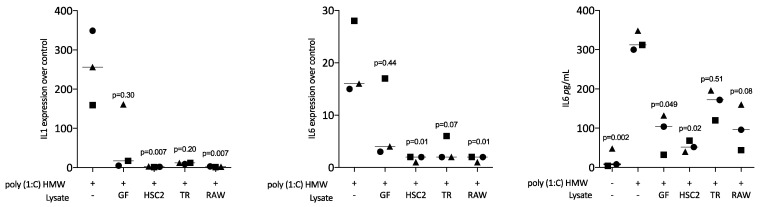
Sonicated necrotic cell lysates reduce TLR3 agonist-induced inflammation of RAW264.7 macrophages. RAW264.7 cells were exposed to necrotic cell lysates from gingival fibroblasts (GF), HSC2, TR146 (TR), and RAW264.7 (RAW) prepared by sonication in the presence of 10 µg/mL poly (1:C) HMW. The data show the x-fold changes in IL1 and IL6 gene expression and the release of IL6 protein into the supernatant. Data points indicate independent experiments. Statistical analysis was based on a Friedmann test and *p*-values are indicated.

Next, we assessed the impact of necrotic cell lysates on poly (1:C) HMW (a TLR3 agonist)-induced inflammation of RAW264.7 macrophages. RT-PCR analyses of IL1, IL6, and an immunoassay of IL6 protein were conducted. LPS caused the expected increased expression of IL1 and IL6 at the transcriptional level, and of IL6 at the protein level. This poly (1:C) HMW response was lowered by necrotic cell lysates from fibroblasts, HSC2, TR146, and RAW264.7 cells (Figure 4). 

### 3.3. Necrotic Cell Lysates Reduce LPS-Induced p65 Nuclear Translocation in RAW264.7 Cells

**Figure 5:**
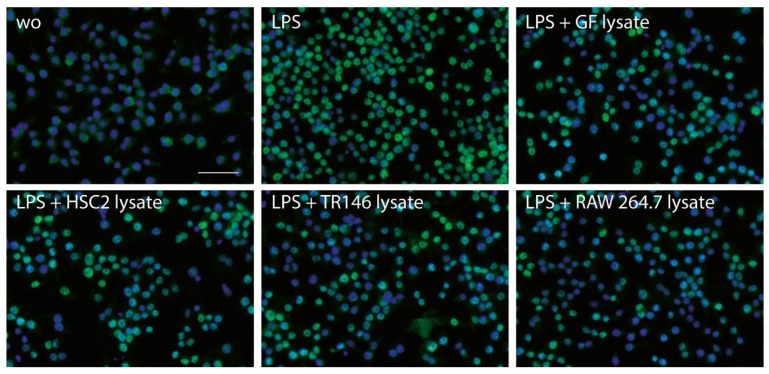
Necrotic cell lysates reduce LPS-induced p65 nuclear translocation in RAW264.7 cells. We used RAW264.7 macrophages exposed to LPS in the presence and absence of necrotic cell lysates from gingival fibroblasts (GF), HSC2, TR146 (TR), and RAW264.7 (RAW) prepared by sonication aiming to induce the nuclear translocation of p65. Immunostaining revealed the green fluorescence signals obtained with the p65 antibody. Note that almost all cells are positive upon LPS treatment, while the cell lysates shift this pattern toward visible blue nuclear staining with DAPI. The scale bar indicates 50 µm.

To further assess how necrotic cell lysates modulate the inflammatory response in RAW264.7 cells, the activation of NFκB-p65 nuclear translocation was performed by immunostaining. In line with the decreased expression of inflammatory markers, necrotic cell lysates prepared by sonication reduced the nuclear translocation of p65 in the presence of necrotic cell lysates (Figure 5). The semiquantitative analysis is presented in Supplementary Table S2. These findings suggest that necrotic cell lysates are capable of reducing the LPS-induced activation of NFκB signaling. 

## 4. Discussion

Tremendous research efforts have been undertaken to uncover the molecular mechanisms of cell death with caspases being the master regulators of apoptosis [11], necroptosis [12], and pyroptosis [13]—all of which cause cell death, thereby provoking a local response. This response can involve inflammation as an early signal to demark cells as damaged and initiate processes aiming to activate tissue homeostasis. In contrast, however, accidental cell death or primary necrosis is a consequence of traumatic events including severe mechanical and thermal stress that culminates in the rupture of cell membranes and the release of cytoplasmatic components [1,2]. It is not astonishing that necrotic cells represent a sign of damage, for instance in trauma or severe burns. It can be assumed that necrotic cells provoke a local inflammatory response by triggering macrophage activation towards the M1 lineage. Surprisingly, however, according to the data provided here, it cannot be ruled out that necrotic cells may have anti-inflammatory activity, indicated by the lowering of the LPS and poly (1:C) HMW-induced expression of IL1 and IL6 in a macrophage cell line.

If we relate our findings to those of others, we have to acknowledge our recent work on necrotic cell lysates prepared from the murine bone marrow mesenchymal cell line ST2 cell and the osteocytic cell line IDG-SW3; these cell lysates clearly show anti-inflammatory activity indicated by the dampening of the LPS-induced macrophage polarization [24]. However, ST2 and IDG-SW3 cell lines do not necessarily represent the necrotic cell damage in the oral cavity; hence, we have extended the original work, now including gingival fibroblasts and the oral squamous carcinoma cell lines HSC2 and TR146 to represent the oral epithelial cell lineage. Consistent with our findings obtained with ST2 and IDG-SW3 cells, necrotic cell lysates from gingival fibroblasts and the oral epithelial cell lines HSC2 and TR146 greatly diminished the LPS as well as the poly (1:C) HMW-induced inflammatory response of RAW264.7 macrophages—and again [24], using sonication, which is more potent compared to freezing/thawing, to prepare the necrotic cell lysates. 

Our data observed with the necrotic lysates from RAW264.7 macrophages were, however, more heterogenous, as, for instance, lysates prepared by freezing/thawing could not reduce the IL6 release of LPS-stimulated macrophages. The reason why we have not measured IL1 is that its release is coupled to pyroptosis, and is thus not ideal to detect IL1 without cell lysis [25]. The dose-response data show a weaker anti-inflammatory activity of RAW264.7 lysates compared to all other cell lysates. Nevertheless, undiluted necrotic lysates from RAW264.7 cells consistently suppressed the LPS and poly (I:C) HMW-induced cytokine expression and p65 nuclear translocation; overall supporting the fundamental observation that necrotic lysates, when prepared by sonication, are robust in their capacity to dampen an inflammatory response of RAW264.7 macrophages. Considering that RAW264.7 macrophages are mouse cells and most of our lysates originate from human cells, future studies might consider a human read-out system with whole blood cells or peripheral blood mononucleated cells. Nevertheless, if we consider the effects of the lysates to be based on binding LPS and poly (I:C) HMW, RAW264.7 cells are fine. Taken together, the data suggest that necrotic lysates from human gingival fibroblasts, HSC2 and TR146 oral epithelial cell lines, and RAW264.7 macrophage cell lines are capable of lowering the agonist-induced activation when using the extremely sensitive murine macrophage cell line.

The question now arises why we have selected those four cell types? Considering that nonsurgical periodontal procedures, such as scaling and root planing [8], as well as electrosurgery [9] and cryosurgery [10], can damage oral epithelial cells and fibroblasts, we have selected cells representing the oral soft tissue. We are asking to what extent and in which direction the local necrotic cells might affect a local inflammatory environment mainly in the soft tissue. We were surprised to learn that all the cell lysates can lower LPS and poly (I:C) HMW-induced inflammatory response in a macrophage cell line. Actually, this paper is a continuation and more of a refinement of our original observation with mouse mesenchymal cells (ST2 bone marrow stromal cells and IDG-SW3 osteocytic cells) [24]. Here, we have mainly focused on oral cells of human origin, thus confirming and strengthening our previous observation. 

The present research raises fundamental questions concerning the molecular mechanism underlying the anti-inflammatory activity observed. Future research should focus on which molecules released cause the observed activity and if this activity is a consequence of the adsorbing and thereby neutralizing of LPS or poly (1:C) HMW, preventing its binding to the TLRs. Potential candidates are clusterin [19], but clusterin [20,21], like uric acid and its salts [22,23], are more of an agonist to M1 macrophages polarization. Extracellular vesicles generated from monocytes under conditions of lytic cell death are also candidates of local cell communication [26]. Consistent with our observations, mesenchymal cell-derived extracellular vesicles prevent the acquisition of the M1 phenotype in LPS-stimulated RAW264.7 macrophages [27,28]. Considering that sonication and freezing/thawing generate extracellular vesicles; these vesicles are potential candidates to mediate anti-inflammatory activity; also knowing that extracellular vesicles may serve for biomedical applications [29]. Moreover, we assume that sonication is more efficient than freezing/thawing to disrupt the membranes; thus, the plasmatic and nuclear fractions are more accessible to the lysates and the RAW264.7 macrophages. We speculate that the biological principle to reduce the inflammatory response is independent of the way to disrupt the cells and, thus, the overall mode of cell necrosis. Consequently, our study remains descriptive in the sense that we cannot explain our findings on a molecular level. Moreover, considering that accidental cell death or primary necrosis [1,2] may occur within the rather invasive dental procedures including implant placement [3,4], hard and soft tissue augmentation [5,7], and all kinds of as scaling and root planing [8] or electrosurgery [9] and cryosurgery [10], our research serves as a primer to further study the potential role of necrotic cells in oral tissue regeneration and homeostasis. 

These findings may inspire further research to translate the in vitro findings towards an in vivo approach; for instance, to embed the respective necrotic cell lysates together with LPS in a matrix that is placed subcutaneously in vivo. Hypothetically, there is less macrophage activation and presumably a diminished immigration of neutrophils to these sites. Another aspect could be related to biomaterial testing, for instance, dental implants and bone substitutes. Macrophages are sensitive to surface modifications underlying the value of macrophages to serve as bioassays for inflammation research [30]. With respect to implant and bone substitute research, we propose to study in vitro how the “coating” of a surface with the sonicated necrotic cell lysates affects the M1 macrophage response, simulating a clinical situation where damaged cells next to the biomaterials may adsorb and potentially affect the local macrophage response. On a tissue level, it would be rewarding to determine signs of primary necrosis in oral tissues upon the various dental treatments and monitor the local inflammatory response. Considering that gingival fibroblasts, but also oral epithelial cells, were identified to be major drivers of inflammation in periodontitis [31], these cell types should be implemented as a bioassay. Interestingly, we have observed that HSC2 and TR147 cell lysates exert a pro-inflammatory activity in gingival fibroblasts (Sordi and Panahipour et al., manuscript submitted). Thus, we have to be careful when interpreting the findings, as necrotic lysates may even increase cytokine expression in other target cells than RAW264.7 macrophages. There is room for future research to study the impact of primary cell necrosis on the overall cellular response, not restricted to inflammation. This is also because the molecules released by cells undergoing primary necrosis may be pharmacologically blocked with a potential beneficial effect on disease outcome [2]. Finally, our observations seem to be a general effect that is not limited to dentistry. This fact should not be considered a limitation, it should rather inspire other fields to focus on the paracrine-like effects of necrotic cells. 

We have to consider the clinical scenario where basically any dental procedure involving instrumentation of tissue will cause an inflammatory response being the forerunner of wound healing. If we consider inflammation as a positive event, which it is, our observations that necrotic cell lysates reduce the M1 response in macrophages obtain a negative flavor. However, it is not easy to put one piece of in vitro observation into the big mosaic of knowledge we have gained from preclinical research and clinical observations. Today, we have to interpret the findings with caution and consider our research as a pilot; an unexpected observation that is seeking its place in the big picture.

## Data Availability

All data are available on demand.

## Supplementary Materials

The following supporting information can be downloaded at: https://www.mdpi.com/article/10.3390/jcm12041701/s1. Figure S1: Sonicated TR146 cell lysates reduce LPS-induced cytokine expression of RAW264.7 cells; Table S1: The effects of the sonicated cell lysates on IL1 and IL6 expression in RAW264.7 macrophages; Table S2: Immunostaining quantification corresponding to Figure 5.
