# Supplementary material for: Oral Cell Lysates Reduce the Inflammatory Response of Activated Macrophages
Source: J Clin Med. 2023 Feb 20;12(4):1701. doi: 10.3390/jcm12041701 (PMC9962209; doi:10.3390/jcm12041701)
Supplement: Supplementary file 1 [file jcm-12-01701-s001.zip › jcm-2160137-supplementary.pdf]

Journal of
Clinical Medicine

MDPI

Supplement File

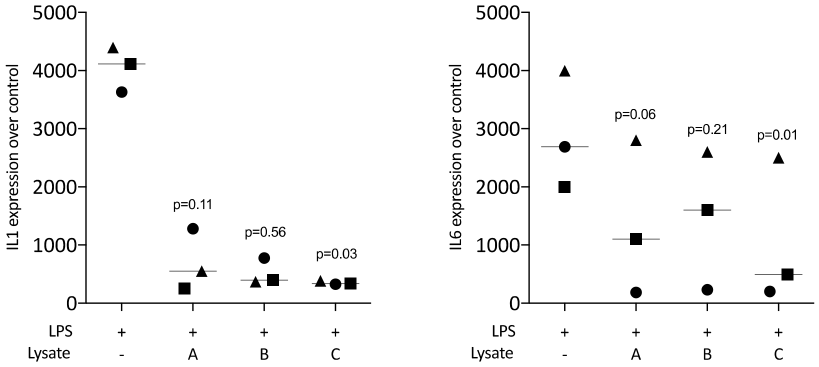

Supplement Figure S1. Sonicated TR146 cell lysates reduce LPS-induced cytokine expression of RAW264.7 cells. (A) RAW264.7 macrophages were pre-exposed to LPS for 15 min before over-night exposure to sonicated TR146 cell lysates. (B) RAW264.7 macrophages were pre-exposed to TR146 cell lysates for 15 min before over-night exposure to LPS. (C) RAW264.7 macrophages were exposed to TR146 cell lysates and LPS simultaneously for over-night. The expression changes of IL1 and IL6 were normalized to an unstimulated control. The bars represent the mean and standard deviation of three independent experiments. Data points indicate independent experiments Statistical analysis was based on a Friedmann test and p-values are indicated.

	IL1 expression mean ± SD	IL6 expression mean ± SD
LPS	392.5 ± 82.5	3241 ± 359
GF lysate	1.05 ± 0.05	0.4 ± 0.1
HSC2 lysate	0.9 ± 0.1	0.2 ± 0.05
TR146 lysate	0.9 ± 0.2	0.3 ± 0.2
RAW 264.7 lysate	1.1 ± 0.05	0.4 ± 0.3

Supplement Table S1. *The effects of the sonicated cell lysates on IL1 and IL6 expression in RAW264.7 macrophages.*
RAW264.7 macrophages were exposed to sonicated cell lysates from gingival fibroblasts (GF), HSC2, TR146 (TR), and RAW264.7 (RAW) cells or LPS. The expression changes of IL1 and IL6 were normalized to an unstimulated control. The Table shows the means and standard deviation (SD) from two independent experiments. The lysates caused no change in IL1 expression and a trend towards lowering of the basal IL6 expression in the macrophages, in contrast to LPS.

	DAPI staining intensity
wo	129.2 ± 2.7
LPS	61.6 ± 2.0
LPS + GF lysate	137.1 ± 6.6
LPS + HSC2 lysate	115.9 ± 23.8
LPS + TR146 lysate	118.8 ± 22.2
LPS + RAW 264.7 lysate	154.9 ± 10.0

Supplement Table S2. *Immunostaining quantification corresponding to Figure 5.*
RAW264.7 macrophages were exposed to LPS in the presence and absence of necrotic cell lysates from gingival fibroblasts (GF), HSC2, TR146 (TR), and RAW264.7 (RAW) prepared by sonication. Nuclear translocation of p65 was visualized by immunostaining and quantified with ImageJ software (see Material and Methods). The data are expressed as arbitrary units representing DAPI blue nuclear staining.
